# Machine learning-based radiomics for bladder cancer staging: evaluating the role of imaging timing in differentiating T2 from T3 disease

**DOI:** 10.3389/fonc.2025.1591742

**Published:** 2025-09-26

**Authors:** Christoph G. Lisson, Luisa Gallee, Konstantin Müller, Sabitha Manoj, Hannah Stöckl, Friedemann Zengerling, Christian Bolenz, Meinrad Beer, Michael Götz, Catharina S. Lisson

**Affiliations:** ^1^ Department of Diagnostic and Interventional Radiology, University Hospital Ulm, Ulm, Germany; ^2^ Artificial Intelligence in Experimental Radiology (XAIRAD), Department of Diagnostic and Interventional Radiology, University Hospital Ulm, Ulm, Germany; ^3^ Department of Urology, University Hospital Ulm, Ulm, Germany; ^4^ Center for Personalized Medicine (ZPM), University Hospital Ulm, Ulm, Germany; ^5^ Division Medical Image Computing, German Cancer Research Center (DKFZ), Heidelberg, Germany

**Keywords:** radiomics, machine learning, bladder cancer, tumor staging, computed tomography, artificial intelligence, image-based biomarkers

## Abstract

**Objectives:**

Accurate preoperative staging of bladder cancer is essential for therapeutic decision-making, particularly in distinguishing between organ-confined (T2) and extravesical (T3) disease. This study aimed to develop a CT-based radiomics model to differentiate T2 from T3 tumors and to evaluate the impact of imaging timing relative to transurethral resection of the bladder (TURB) on model performance. Additionally, we assessed the added diagnostic value of integrating routine clinical biomarkers.

**Methods:**

In this retrospective study, 97 patients with histologically confirmed bladder cancer who underwent TURB followed by contrast-enhanced CT were included. Tumor segmentation was performed using a semi-automated three-dimensional approach, and radiomic features were extracted according to IBSI standards. A random forest classifier was trained to distinguish between T2 and T3 tumors. Patients were stratified according to the interval between TURB and CT imaging (≤14 days vs >14 days). Performance metrics were assessed for both radiomics-only and combined clinical-radiomics models. Clinical variables included preoperative creatinine, hemoglobin, arterial hypertension, diabetes mellitus, smoking status, and tumor size.

**Results:**

The radiomics-only model achieved an AUC of 0.68 in Cohort 1 (≤14 days post-TURB). In Cohort 2 (>14 days post-TURB), model performance improved with an AUC of 0.80. The combined clinical-radiomics model further enhanced performance, yielding an AUC of 0.76 in Cohort 1 and 0.82 in Cohort 2. Delayed imaging was associated with increased radiomic feature stability and improved classification accuracy, suggesting a potential benefit of temporal separation from post-surgical tissue changes.

**Conclusion:**

This study demonstrates the feasibility of CT-based radiomics using full-volume 3D tumor segmentation to distinguish between T2 and T3 bladder cancer. The integration of clinical biomarkers and consideration of imaging timing significantly improved model performance. These findings support the development of temporally optimized, multimodal prediction models for individualized bladder cancer staging and treatment planning.

## Introduction

Urothelial carcinoma (UC), commonly known as bladder cancer (BCa), is the 10th most common cancer worldwide, with approximately 500,000 new cases and 200,000 deaths each year (1).

Tobacco smoking is the primary risk factor, accounting for roughly 50% of cases, followed by occupational exposure to aromatic amines and ionizing radiation (van 2, 3).

Painless hematuria is the most common initial symptom and warrants thorough evaluation in all cases (4).

Approximately 75% of bladder cancer patients present with non-muscle invasive bladder cancer (NMIBC), classified as stage pTa, pT1, or carcinoma *in situ* (pTis). In contrast, the majority of muscle-invasive bladder cancer (MIBC) cases—stages pT2a to pT4b—are diagnosed as primary invasive disease, although up to 15% of MIBC patients have a history of high-risk NMIBC. All cases of MIBC are considered high grade (5).

Muscle-invasive bladder cancer (MIBC) is categorized into stages T2, T3, and T4 based on the extent of tumor infiltration. In T2, the tumor invades the detrusor muscle; in T3, it extends into the perivesical fat; and in T4, it breaches into adjacent organs such as the prostate, uterus, or pelvic wall. The depth of invasion serves as a critical prognostic factor and is pivotal in guiding treatment strategies for localized bladder cancer (5).

The clinical management of muscle-invasive bladder cancer (MIBC) is primarily guided by the tumor’s T stage, as the risk of lymph node metastasis increases with more advanced local tumor progression. This stratification necessitates tailored treatment approaches. For instance, patients with clinical T2 (cT2) disease may be considered for partial cystectomy in combination with neoadjuvant cisplatin-based chemotherapy (6). In contrast, patients diagnosed with cT3 or cT4a disease are typically managed with more aggressive treatments, which may include radical cystectomy, radiation therapy, chemotherapy, immunotherapy, or a combination of these modalities, depending on the specific stage and clinical context (4).

Transurethral resection of bladder tumor (TURBT), followed by pathological analysis, is essential for diagnosing, staging, and managing bladder cancer (7). However, TURBT has notable limitations in assessing muscle layer involvement; studies have shown that up to 50% of patients initially staged as T1 are later found to have muscle-invasive disease at the time of radical cystectomy (8).

Therefore, a comprehensive evaluation of the entire urothelium is crucial for detecting synchronous secondary tumors (4). Multiphasic contrast-enhanced computed tomography (CT), including CT urography, is recommended for this purpose (9).

Magnetic resonance imaging (MRI) has become increasingly important for the local staging of bladder cancer, especially when differentiating early-stage tumors (10). Functional MRI techniques, notably diffusion-weighted imaging (DWI) and dynamic contrast-enhanced MRI (DCE-MRI), have demonstrated potential in distinguishing non-muscle-invasive (T1) from deep muscle-invasive (T2b) disease—a distinction that is critical for guiding therapeutic decisions. However, accurately identifying muscle-invasive (T2) and microscopic extravesical (T3a) disease remains challenging. For more advanced stages, such as T3b and T4 disease, both computed tomography (CT) and MRI play essential roles in comprehensive assessment (10).

In the present study, we chose contrast-enhanced CT as the radiological basis for radiomic feature extraction. This decision was driven by CT’s widespread clinical availability, its role as the standard imaging modality in global bladder cancer staging protocols, and its routine preoperative use in many urologic centers. MRI was not included due to limited institutional availability and non-standardized protocols at the time of data collection. Importantly, our aim was to establish radiomics feasibility in a clinically realistic and widely generalizable setting. CT-based radiomics thus provides a pragmatic foundation for subsequent multimodal imaging studies.

Recent advances in machine learning, coupled with increasing computational capacity, have accelerated the development of radiomics as a quantitative imaging discipline (11, 12). Radiomics enables the extraction of high-dimensional, quantifiable features from medical images—particularly of tumors—to characterize tissue heterogeneity, morphology, and signal intensity patterns (13–15). These features are subsequently processed using machine learning or deep learning algorithms to build predictive models that can assist and refine clinical decision-making, especially in oncologic contexts.

In the context of bladder cancer, several studies have demonstrated the feasibility of radiomics and deep learning models to predict clinically relevant parameters such as preoperative tumor grade, lymph node metastases, or the presence of muscle-invasive disease using CT or MRI-based features (16–19).

However, these investigations have primarily addressed the general dichotomy between non-muscle-invasive (≤T1) and muscle-invasive (≥T2) stages, without focusing on more granular and clinically decisive stage distinctions.

To date, no study has systematically examined whether radiomics can differentiate T2 (organ-confined, intravesical) from T3 (extravesical, perivesical fat infiltration) bladder cancer based on CT imaging, despite the high clinical relevance of this boundary for surgical planning and prognostic assessment.

Moreover, another critical yet underexplored variable is the timing of imaging relative to transurethral resection of the bladder (TURB)—a factor that may substantially influence imaging characteristics due to inflammatory changes, edema, or early tissue remodeling, particularly in the perivesical region.

None of the existing radiomics studies have investigated how such temporal variation might affect the accuracy or stability of AI-driven staging models.

Our study addresses both of these previously unexplored dimensions. Specifically, we present the first CT-based machine learning model capable of distinguishing between T2 and T3 tumors, thereby providing staging information that directly informs therapeutic decision-making.

In addition, by analyzing patient cohorts with defined intervals between TURB and staging CT, we systematically evaluate the impact of imaging timing on model performance. This approach not only reflects common real-world diagnostic pathways but also provides insight into the temporal robustness of radiomic signatures.

By integrating radiomics with clinical parameters in a hybrid model, we further enhance staging accuracy, particularly in patients with delayed post-TURB imaging. Collectively, these methodological innovations represent a significant step toward personalized, image-based treatment stratification in bladder cancer.

## Materials and methods

### Patients

We retrospectively identified 133 patients with localized bladder cancer, confirmed by pathological diagnosis after surgical resection, from our hospital database between 2012 and 2020. Only patients who had undergone a standard contrast-enhanced CT scan of the abdomen and pelvis before surgery were included (n = 105).

To ensure a sufficient lesion area for drawing regions of interest (ROI), we excluded patients with tumors smaller than 5 mm, those with bladder wall thickening without a distinct mass, and those with insufficient imaging quality due to artifacts from metal implants or motion.

The final study cohort consisted of 97 patients, categorized into intravesical (≤T2) and extravesical (≥T3) disease.

Clinical information, including patient age, sex, and pathological stage, was retrospectively retrieved from electronic health records. Histopathological classification was based on the 2016 WHO criteria (20).

Patients were included in the study if they met the following criteria: (1) pathologically confirmed urothelial carcinoma, (2) underwent radical cystectomy (RC), and (3) received a standard contrast-enhanced CT scan of the abdomen and pelvis within 30 days before surgery.

Patients were excluded if they met one or more of the following criteria: (1) prior neoadjuvant chemotherapy or preoperative radiotherapy, (2) concurrent malignancies known at time of CE, (3) imaging artifacts precluding reliable tumor segmentation, or (4) incomplete or missing clinical and/or imaging data.

The study was approved by the institutional review board (protocol number 378/24), and the requirement for written informed consent was waived.

**Figure 1:**
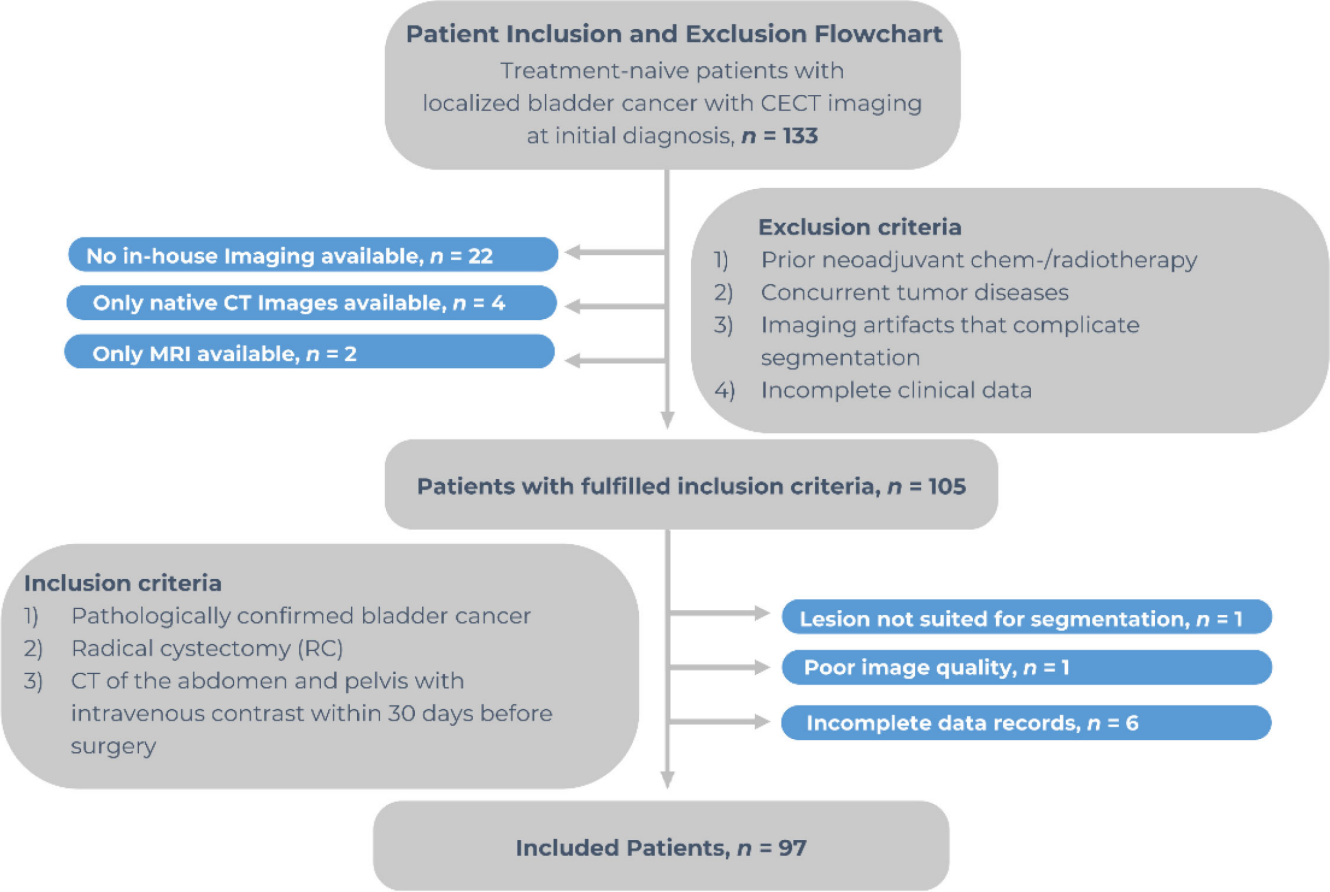
Recruitment pathway of the study.

The patient recruitment process is illustrated in **Figure 1**.

### Image acquisition

All patients underwent contrast-enhanced CT scans according to standard clinical protocols for routine staging. Imaging was performed before surgery as part of the routine staging procedure to assess disease status. For image segmentation and analysis, all reconstructed images were retrieved from the hospital’s picture archiving and communication system (PACS).

### Statistics for clinical characteristics

To test for differences in the clinical characteristics between the two groups ≤T2 (intravesical disease) and ≥T3 (extravesical disease), Pearson’s chi-square test was applied for categorical variables and the independent samples t-test for continuous variables. In cases of unequal variances (tested with Levene’s test), the t-test results were adjusted accordingly. All analyses were conducted using IBM SPSS Statistics for Windows, Version 29.0 (IBM Corp., Armonk, NY, USA).

### ROI-segmentation and imaging feature extraction

The evaluation of imaging features, such as histogram features and those derived from co-occurrence matrices, was first introduced by Haralick et al. in 1973 (21) and has since demonstrated substantial potential across various cancer types and clinical applications (22, 23). In this study, three-dimensional region-of-interest (ROI) segmentation, texture analysis, and feature extraction were conducted using Mint Lesion™ software (version 3.8.4, mint Medical GmbH, Heidelberg, Germany).

Mint Lesion™ is a specialized medical software platform that facilitates the analysis, 3D visualization, and comparison of radiological images from modalities such as CT, MRI, and PET. It supports radiologists in both clinical evaluations and research, allowing for seamless image import from PACS and structured report export to systems such as PACS, RIS/HIS, or study management platforms. The software is classified as a Class IIb medical device, certified under EU Regulation 2017/745 (Medical Device Regulation, MDR). Its CE marking (CE 0123) confirms compliance with the General Safety and Performance Requirements of the MDR. Details of the feature extraction settings are provided in **Supplementary Table S1**.

Image analysis was performed by two board-certified radiologists, each with over 10 years of experience in oncological imaging and at least 8 years of expertise in texture analysis. Radiomic features were quantified by analyzing distinct grey-level patterns within the ROIs, with texture feature descriptors generated in accordance with the Image Biomarker Standardisation Initiative (IBSI) guidelines (24).

A total of 77 imaging features were calculated for each ROI, encompassing tumor size and shape in three dimensions. Additionally, first-order statistics were used to describe the distribution of voxel intensities within the ROI. To capture voxel intensity patterns, texture-based features were derived from the grey-level co-occurrence matrix (GLCM). Additional details can be found in **Supplementary Tables S1** and **S2**, available in the **Supplementary Materials**. The extracted 3D volumetric radiomic features served as input data for machine learning model development.

### Feature selection

After preprocessing, feature selection was performed using the Random Forest algorithm. As in other data-mining applications, radiomics is affected by the curse of dimensionality (25), as it involves extracting a vast number of quantitative features from regions of interest (ROIs). Implementing an appropriate feature selection strategy is crucial to reduce the dimensionality of radiomic data.

By selecting an optimal subset of features, overfitting is minimized, resulting in models with improved generalizability, greater simplicity, faster computation, and enhanced predictive performance (26).

Filter methods are widely used for feature selection and can be categorized based on the criteria they employ, such as dependence, similarity, and other statistical measures. These methods assess the relevance of individual features independently of the learning model, typically using metrics such as correlation coefficients, mutual information, or statistical tests.

By preselecting informative features before model training, filter methods help reduce dimensionality, improve computational efficiency, and enhance model interpretability while mitigating the risk of overfitting (27, 28).

Random Forest is an ensemble learning method that constructs multiple decision trees using randomly selected subsets of data and features, with predictions averaged across all trees. In feature selection, Random Forest can function as a filter method by assessing the importance of each feature using metrics such as Gini impurity or information gain. This approach enables the identification and removal of less relevant features before model training, improving both model performance and interpretability (29). In this study, feature selection was performed using the Weka Toolkit (version 3.8), a widely used machine learning software that provides various algorithms for data preprocessing, feature selection, and model evaluation (30).

To ensure the stability and interpretability of our machine learning model, we conducted a multicollinearity analysis by calculating the Variance Inflation Factor (VIF) for all radiomic and clinical features. Features with a VIF greater than 10 were considered highly collinear and were excluded from further analysis, in line with established statistical recommendations. This filtering step improved the selection of independent, informative features for model training and reduced the risk of redundancy-driven overfitting (31–33).

Following VIF-based feature selection (threshold: VIF < 10), we generated heatmaps to visualize pairwise Pearson correlation coefficients among the retained features. In total, 61 radiomic features exceeded the predefined VIF threshold and were excluded from further analysis, while 23 features with acceptable multicollinearity levels were retained for model development (see **Supplementary Tables S3** and **S4**).

These heatmaps served to verify that the selected radiomics and clinical features exhibited minimal linear interdependencies. Strong positive or negative correlations—depicted by dark red or blue hues—were rare across the filtered feature sets. In particular, the clinical variables (panels b and d) demonstrated consistently low intercorrelation levels, as indicated by light, near-neutral tones in the upper and left matrix sections.

**Figure 2:**
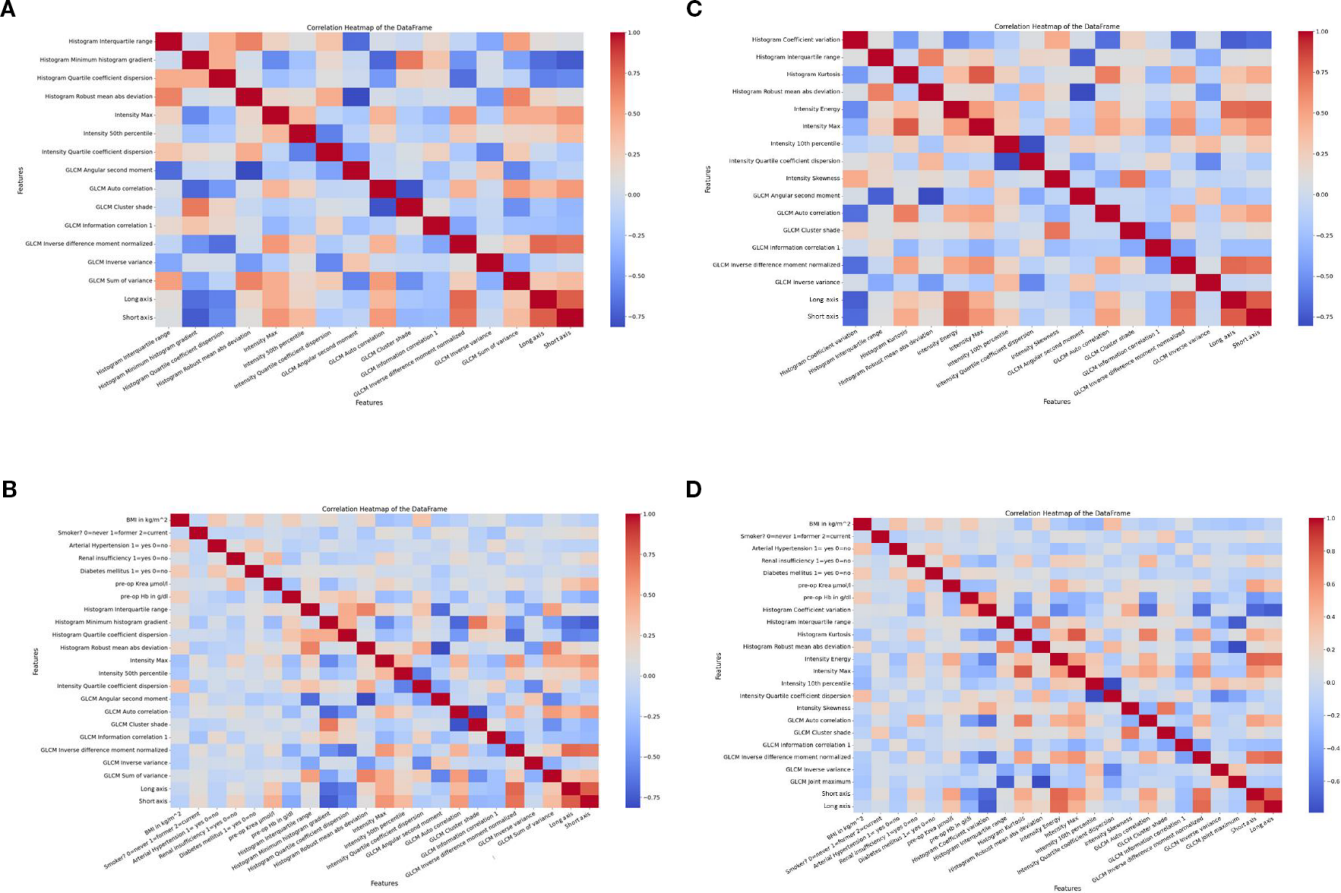
The heatmaps visualize the relationships between extracted features, highlighting clusters and correlations. This helps identify feature dependencies and potential redundancies. **(a)** Heatmap at delay 0 for radiomics features; **(b)** Heatmap at delay 0 for clinical and radiomics features. The heatmaps visualize the relationships between extracted features, highlighting clusters and correlations. This helps identify feature dependencies and potential redundancies. **(c)** Heatmap at delay 14 for radiomics features; **(d)** Heatmap at delay 14 for clinical and radiomics features.

To further assess the potential impact of imaging timing on inter-feature correlations, separate heatmaps were constructed for both patient subgroups: those undergoing immediate imaging (delay 0) and those with delayed imaging (delay ≥14 days). Within each subgroup, distinct heatmaps were generated for the radiomics-only features and the combined clinical-radiomics feature sets. For details, see the heatmaps in **Figures 2a–d**.

This stratified visualization allowed for a more differentiated analysis of temporal variability in correlation patterns and potential redundancies across feature domains.

Taken together, the heatmaps complemented the VIF-based multicollinearity analysis by enabling a qualitative inspection of correlation structures. The overall low degree of linear correlation among retained features confirms the effectiveness of our collinearity filtering strategy and underscores the robustness of the final feature set used for model development. This methodological approach enhances the interpretability, reproducibility, and potential clinical applicability of our radiomics model. (34).

### Development and validation of predictive models for tumor infiltration assessment

In this study, we employed the Random Forest (RF) algorithm, a well-established machine learning technique, to develop an optimal model for distinguishing between muscle-invasive (T2) and extravesical (T3) disease in bladder cancer.

RF-based methods provide a robust and efficient alternative to deep learning models in medical imaging, offering comparable performance without the need for extensive computational resources (35). The effectiveness and applicability of RF in medical imaging have been extensively documented in the literature (36–40).

To optimize the model’s performance and maximize the area under the receiver operating characteristic curve (AUC-ROC), we fine-tuned hyperparameters using a grid search procedure (41). The optimal settings identified were max_depth = 8 and criterion = ‘gini’.

Robustness was ensured through fivefold cross-validation. Clinical parameters incorporated into the analysis included smoking status, arterial hypertension, diabetes mellitus, preoperative creatinine, preoperative hemoglobin and tumor size, as these have been identified in the literature as potential risk factors for bladder cancer (4, 42; van 43–48).

A total of 97 patients were included in the study. To investigate the effect of imaging timing on model performance, we defined two cohorts: Cohort 1 comprised the entire patient population regardless of the interval between transurethral resection of the bladder (TURB) and CT imaging, while Cohort 2 consisted of a subset of 79 patients who underwent CT at least 14 days after TURB. For both cohorts, the dataset was split into training and test sets using a 70:30 ratio. In Cohort 1, 67 patients were assigned to the training set and 30 to the test set. In Cohort 2, 55 patients were included in the training set and 24 in the test set.

For each cohort, we constructed two types of models:

1. Radiomics-only model: utilizing solely radiomic features extracted from imaging data.
2. Combined radiomics-clinical model: integrating radiomic features with relevant clinical data.

The performance of both models was evaluated using receiver operating characteristic (ROC) curve analysis, with standard deviations and confidence intervals calculated.

To complement the overall assessment of classification performance, we performed subgroup analyses stratified by gender and age at initial diagnosis. For the age-based analysis, patients were categorized into two groups: those older than 70 years and those aged 70 years or younger. These stratifications aimed to evaluate potential differences in model performance related to gender and age.

In Cohort 1, which included all patients regardless of the timing of their transurethral resection of the bladder (TURB), the gender-specific distribution was as follows: 55 male patients (Gender = 1) were assigned to the training set and 25 to the test set, while 12 female patients (Gender = 2) were included in the training set and 5 in the test set. In Cohort 2, which included only patients who underwent TURB at least 14 days prior to imaging, the gender-specific subsets consisted of 45 male patients in the training set and 19 in the test set, and 10 female patients in the training set and 5 in the test set.

With respect to age, in Cohort 1, the subgroup of patients older than 70 years comprised 52 individuals in the training set and 23 in the test set, while the subgroup aged 70 years or younger included 15 individuals in the training set and 7 in the test set. In Cohort 2, 43 patients older than 70 years were assigned to the training set and 18 to the test set, whereas 12 patients aged 70 years or younger were included in the training set and 6 in the test set.

These stratified analyses allowed for a more nuanced evaluation of model robustness and generalizability across clinically relevant subgroups and facilitated the identification of potential performance disparities associated with gender or age.

To assess clinical utility, decision curve analysis (DCA) was performed. This method evaluates the net benefit of predictive models across different threshold probabilities in the training population, enabling a direct comparison of model performance in terms of clinical relevance and decision-making impact. Feature selection and model construction were implemented using the open-source Python machine learning library Scikit-learn (Python version 3.10, Scikit-learn version 0.23.3, http://scikit-learn.org/) (49, 50) (see **Supplementary Table 5** for details).

## Results

### Patient characteristics

The study included 97 consecutive patients with histologically confirmed bladder cancer (mean age: 68.8 ± 10.5 years, range: 39 – 89). Among these, 51 patients (52.6%) presented with extravesical (≥T3) disease in muscle-invasive bladder cancer (MIBC).

There were no statistically significant differences in the following clinical characteristics between patients with muscle-invasive (T2) and extravesical disease (T3) based on Pearson’s chi-square test: average age, sex, weight, height, BMI, arterial hypertension, cardiovascular disease, renal insufficiency, diabetes mellitus, or smoking status (former/current).

Statistically significant differences were observed in the clinical characteristics preoperative creatinine and preoperative hemoglobin between patients with muscle-invasive (T2) and extravesical disease (T3) based on T-test (p < 0.05).

We investigated the ability of our model to differentiate ≤T2 vs. ≥T3 across two cohorts:

- Cohort 1: Included all patients, irrespective of the timing of their transurethral resection of the bladder (TURB) (mean 22.33 days delay, range 5.475 – 39.185)
- Cohort 2: Comprised patients who underwent TURB at least 14 days prior imaging (d > 14; mean 26.43 days delay, range 15.07 – 37.79).

The clinical characteristics of cohort 1 and 2 are summarized in **Tables 1** and **2**.

**Table 1:** The clinical characteristics of the patients in cohort 1.

Characteristic	≤T2 (intravesical disease)	≥T3 (extravesical disease)	p-value (Pearson’s chi-square test for categorical variables; independent samples t-test for continuous variables). The symbol ** indicates statistically significant p-values.
Number of patients (*n*)	46 (47 %)	51 (53 %)	
Average age (mean)	67.28 ± 9.30 years	70.08 ± 11.41 years	0.192
Sex			1.000
Male	38 (83 %)	42 (82 %)	
Female	8 (17 %)	9 (18 %)	
Weight (kg) (mean)	85.54 ± 16.84	79.18 ± 14.24	0.047**
Hight [cm] (mean)	174.57 ± 0.08	173.69 ± .08	0.587
BMI [kg/m²] (mean)	27.94 ± 4.52	26.14 ± 4.15	0.044**
pre-op Krea [µmol/l] (mean)	90.35 ± 22.11	122.24 ± 88.66	0.016**
pre-op Hb [g/dl] (mean)	13.80 ± 1.93	12.39 ± 2.41	0.002**
Arterial Hypertension	32 (70 %)	33 (65 %)	0.669
Cardiovascular Disease	11 (24 %)	15 (29 %)	0.648
Renal insufficiency	6 (13 %)	10 (20 %)	0.424
Diabetes mellitus	8 (17 %)	11 (22 %)	0.621
Smoker	17 (37 %)	20 (39 %)	0.082

**Table 2:** The clinical characteristics of the patients in cohort 2.

Characteristic	≤T2 (intravesical disease)	≥T3 (extravesical disease)	p-value (Pearson’s chi-square test for categorical variables; independent samples t-test for continuous variables). The symbol ** indicates statistically significant p-values.
Number of patients (*n*)	39 (49 %)	40 (51 %)	
Average age (mean)	67.44 ± 9.51 years	71.60 ± 10.67 years	0.071
Sex			1.000
Male	31 (79 %)	33 (83 %)	
Female	8 (21 %)	7 (18 %)	
Weight (kg) (mean)	85.54 ± 16.84	79.18 ± 14.24	0.081
Hight [cm] (mean)	174.10 ± .082	173.83 ± .081	0.879
BMI [kg/m^2] (mean)	28.15 ± 4.70	26.14 ± 4.59	0.057
pre-op Krea [µmol/l](mean)	88.56 ± 18.59	123.50 ± 97.78	0.032**
pre-op Hb [g/dl] (mean)	14.033 ± 1.66	12.29 ± 2.36	<0.001**
Arterial Hypertension	26 (67 %)	26 (65 %)	1.000
Cardiovascular Disease	9 (23 %)	12 (30 %)	0.612
Renal insufficiency	6 (15 %)	9 (23 %)	0.568
Diabetes mellitus	7 (18 %)	9 (23 %)	0.781
Smoker (former/current)	12 (31 %)	16 (40 %)	0.141

### Radiomics model development: feature selection and performance evaluation

**Figure 3:**
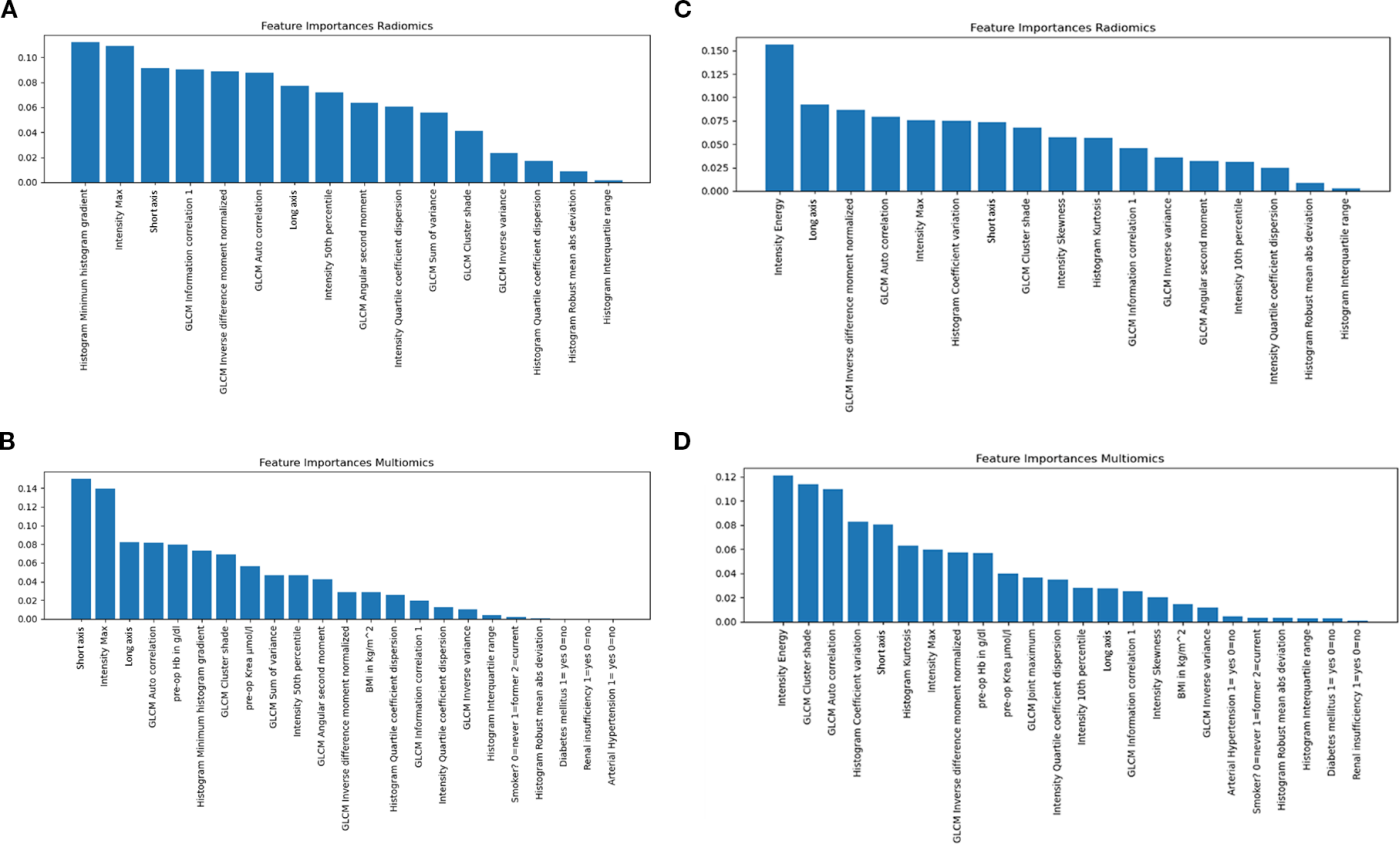
The Feature Importance Plots (extraction setting: resample kein_filter) visually represent the contribution of individual radiomics and clinical features to the predictive performance of the Random Forest (RF) model for tumor invasion extent. **(a)** Feature Importance Plot at delay 0 for radiomics features; **(b)** Feature Importance Plot at delay 0 for radiomics and clinical features. The Feature Importance Plots (extraction setting: resample kein_filter) visually represent the contribution of individual radiomics and clinical features to the predictive performance of the Random Forest (RF) model for tumor invasion extent. **(c)** Feature Importance Plot at delay 14 for radiomics features; **(d)** Feature Importance Plot at delay 14 for radiomics and clinical features.

The dataset comprised 97 sample instances, each representing bladder cancer as the volume of interest in an individual patient. Of these, 51 instances belonged to the “≥T3 extravesical disease” category, while 46 instances were in the “≤T2 intravesical disease” category. A total of 77 radiomic features were extracted from venous-phase CT images of the training cohort. Additional details can be found in **Supplementary Tables S1** and **S2**, available in the **Supplementary Materials**. Using the Random Forest algorithm for feature screening, the 35 most important radiomic features were selected as the best-performing predictors for bladder wall invasion (for details, see the feature importance plots in **Figure 3**).

We evaluated the ability of our model to differentiate between ≤T2 and ≥T3 across two cohorts. Cohort 1 included all patients irrespective of TURB timing relative to CT, regardless of the timing of their transurethral resection of the bladder (TURB) (d = 0, mean 22.33 days delay, range 5.475 - 39.185). Cohort 2 comprised those with TURB at least 14 days before imaging (d > 14; mean 26.43 days delay, range 15.07 - 37.79). These features were used as input for the machine learning-based radiomics modeling for both cohorts. Standard evaluation metrics for machine learning, including accuracy, precision, F1-score, and the area under the ROC curve (AUC), were applied to assess the models' performance in predicting the extent of tumor invasion. All statistical tests were two-sided, and a p-value < 0.05 was considered statistically significant.

In the ROC analysis of the radiomics models, classification metrics obtained from fivefold cross-validation were as follows: an AUC of 0.68 (± 0.08), accuracy 0.63 ± 0.09, precision 0.63 ± 0.10, recall 0.63 ± 0.09, F1-score 0.62 ± 0.10, sensitivity 0.74 ± 0.12 and specificity 0.58 ± 0.17 for Cohort 1; and an AUC of 0.80 ± 0.08, accuracy 0.73 ± 0.09, precision 0.75 ± 0.10, recall 0.73 ± 0.09, F1-score 0.72 ± 0.09, sensitivity 0.80 ± 0.08 and specificity 0.63 ± 0.11 for Cohort 2.

In comparison, the combined model in Cohort 1 --which integrated clinical risk factors with radiomic features --achieved improved performance with an AUC of 0.76 ± 0.09, accuracy 0.69 ± 0.07, precision 0.70 ± 0.08, recall 0.68 ± 0.07, F1-score 0.68 ± 0.07, sensitivity 0.74 ± 0.14 and specificity 0.62 ± 0.16.

These results indicate that the inclusion of clinical variables can enhance the predictive performance of radiomics-based models in preoperative bladder cancer staging.

A similar pattern was observed in Cohort 2. While the radiomics-only model yielded strong metrics, the combined clinical-radiomics model demonstrated further gains, achieving an AUC of 0.82 ± 0.07, accuracy 0.78 ± 0.05, precision 0.79 ± 0.05, recall 0.78 ± 0.05, F1-score 0.77 ± 0.05, sensitivity 0.80 ± 0.08 and specificity 0.63 ± 0.11. These results underscore the benefit of integrating clinical variables into radiomic models, especially in temporally optimized imaging settings.

The predictive performances of the radiomics-only and combined clinical-radiomics models in both cohorts are summarized in **Tables 3** and **4**.

**Table 3:** The predictive performances of the radiomics-only and combined clinical-radiomics models in cohort 1 including all patients, irrespective of the timing of their transurethral resection of the bladder (TURB).

Model	AUC (mean ± SD)	Accuracy (mean ± SD)	Precision (mean ± SD)	Recall (mean ± SD)	F1 Score (mean ± SD)	Sensitivity (mean ± SD)	Specificity (mean ± SD)
Radiomics-only	0.68 ± 0.08	0.63 ± 0.09	0.63 ± 0.10	0.63 ± 0.09	0.62 ± 0.10	0.74 ± 0.12	0.58 ± 0.17
Combined Clinical-Radiomics	0.76 ± 0.09	0.69 ± 0.07	0.70 ± 0.08	0.68 ± 0.07	0.68 ± 0.07	0.74 ± 0.14	0.62 ± 0.16

**Table 4:** Predictive performance of the radiomics-only and combined clinical-radiomics models in cohort 2, which includes patients who underwent TURB at least 14 days before imaging.

Model	AUC (mean ± SD)	Accuracy (mean ± SD)	Precision (mean ± SD)	Recall (mean ± SD)	F1-Score (mean ± SD)	Sensitivity (mean ± SD)	Specificity (mean ± SD)
Radiomics-only	0.80 ± 0.08	0.73 ± 0.09	0.75 ± 0.10	0.73 ± 0.09	0.72 ± 0.09	0.69 ± 0.10	0.71 ± 0.12
Combined Clinical-Radiomics	0.82 ± 0.07	0.78 ± 0.05	0.79 ± 0.05	0.78 ± 0.05	0.77 ± 0.05	0.80 ± 0.08	0.63 ± 0.11

**Figure 4:**
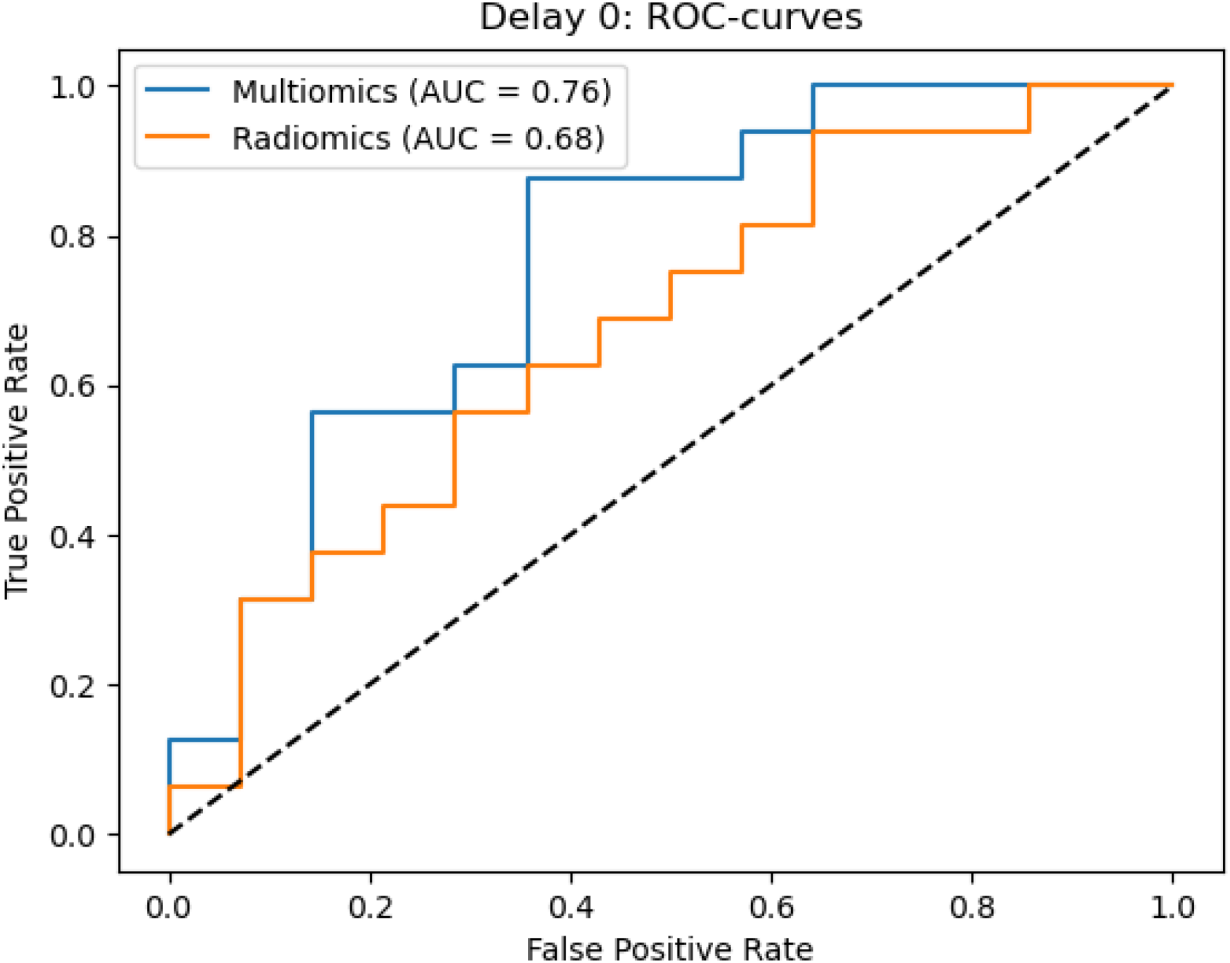
Receiver operating characteristic (ROC) curves for the radiomics-only and combined clinical-radiomics models in cohort 1, showing that the combined model slightly outperforms the radiomics-only approach in predicting bladder wall invasion.

**Figure 5:**
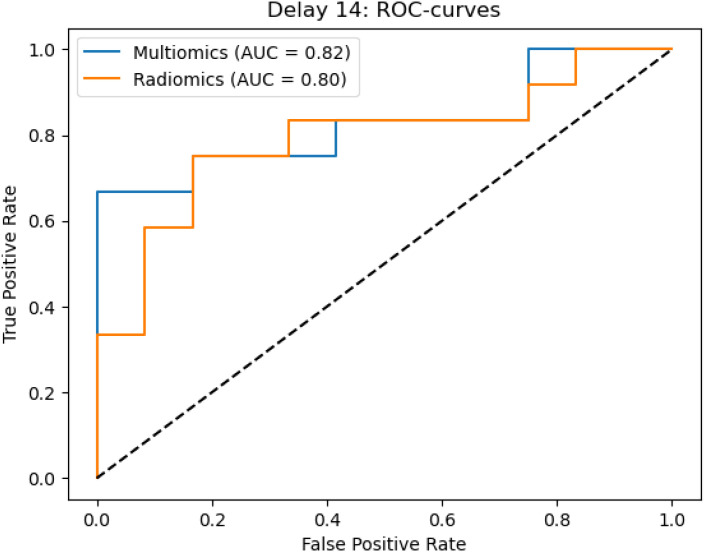
ROC curves for cohort 2, demonstrating improved performance of the combined clinical-radiomics model compared to the radiomics-only model in predicting bladder wall invasion.

The ROC curves highlight the predictive performance of the radiomics-only and combined clinical-radiomics models. Cohort 1 included all patients, irrespective of TURB timing relative to CT, whereas Cohort 2 comprised those with TURB at least 14 days before imaging. The combined model consistently outperforms the radiomics-only approach, achieving higher AUC values and improving discrimination between ≤T2 and ≥T3 stages. For details, see the ROC curves in **Figures 4** and **5**.

To further investigate the model’s performance across different patient subgroups, we conducted gender-specific analyses by calculating sensitivity and specificity separately for male and female patients.

Among male patients, the radiomics-only model in Cohort 1 yielded a sensitivity of 0.72 (± 0.13) and a specificity of 0.58 (± 0.14). The combined clinical-radiomics model showed a modest improvement, achieving a sensitivity of 0.73 (± 0.14) and a specificity of 0.61 (± 0.15). In Cohort 2, the radiomics-only model produced a sensitivity of 0.72 (± 0.15) and a specificity of 0.70 (± 0.13), while the addition of clinical parameters further enhanced performance, reaching a sensitivity of 0.78 (± 0.14) and a specificity of 0.72 (± 0.12).

Among female patients, the radiomics-only model in Cohort 1 yielded a sensitivity of 0.67 (± 0.22) and a specificity of 0.65 (± 0.24). The combined model improved both metrics, with a sensitivity of 0.77 (± 0.27) and a specificity of 0.70 (± 0.35). In Cohort 2, the radiomics-only model achieved a balanced performance with a sensitivity and specificity of 0.70 (± 0.26) and 0.70 (± 0.11), respectively.

Notably, the combined model in this subgroup demonstrated reduced performance, with a sensitivity of 0.53 (± 0.23) and specificity of 0.60 (± 0.39). This observation may reflect underlying sex-specific differences in tumor biology or image-derived patterns and underscores the need for further research into gender-informed modeling strategies.

In addition, a subgroup analysis was performed based on age at initial diagnosis, stratifying patients into two groups: >70 years and ≤70 years. Among patients older than 70 years, the radiomics-only model in Cohort 1 yielded a sensitivity of 0.55 (± 0.16) and a specificity of 0.72 (± 0.12), whereas the combined clinical-radiomics model improved sensitivity to 0.64 (± 0.15) and specificity to 0.74 (± 0.17). In Cohort 2, sensitivity and specificity increased from 0.58 (± 0.14) and 0.84 (± 0.12) with the radiomics-only model to 0.63 (± 0.17) and 0.79 (± 0.10), respectively, with the combined model.

For patients aged ≤70 years, the radiomics-only model in Cohort 1 demonstrated a sensitivity of 0.67 (± 0.35) and a specificity of 0.60 (± 0.34). The addition of clinical parameters improved performance, yielding a sensitivity of 0.70 (± 0.25) and a specificity of 0.70 (± 0.20). In Cohort 2, the radiomics-only model achieved a sensitivity of 0.73 (± 0.26) and a specificity of 0.67 (± 0.22), while the combined model further improved sensitivity to 0.87 (± 0.17) and maintained a specificity of 0.67 (± 0.31).

Taken together, these findings suggest that integrating clinical features consistently enhances model performance across both age groups, with particularly pronounced gains in sensitivity in younger patients and improvements in specificity among older individuals.

**Figure 6:**
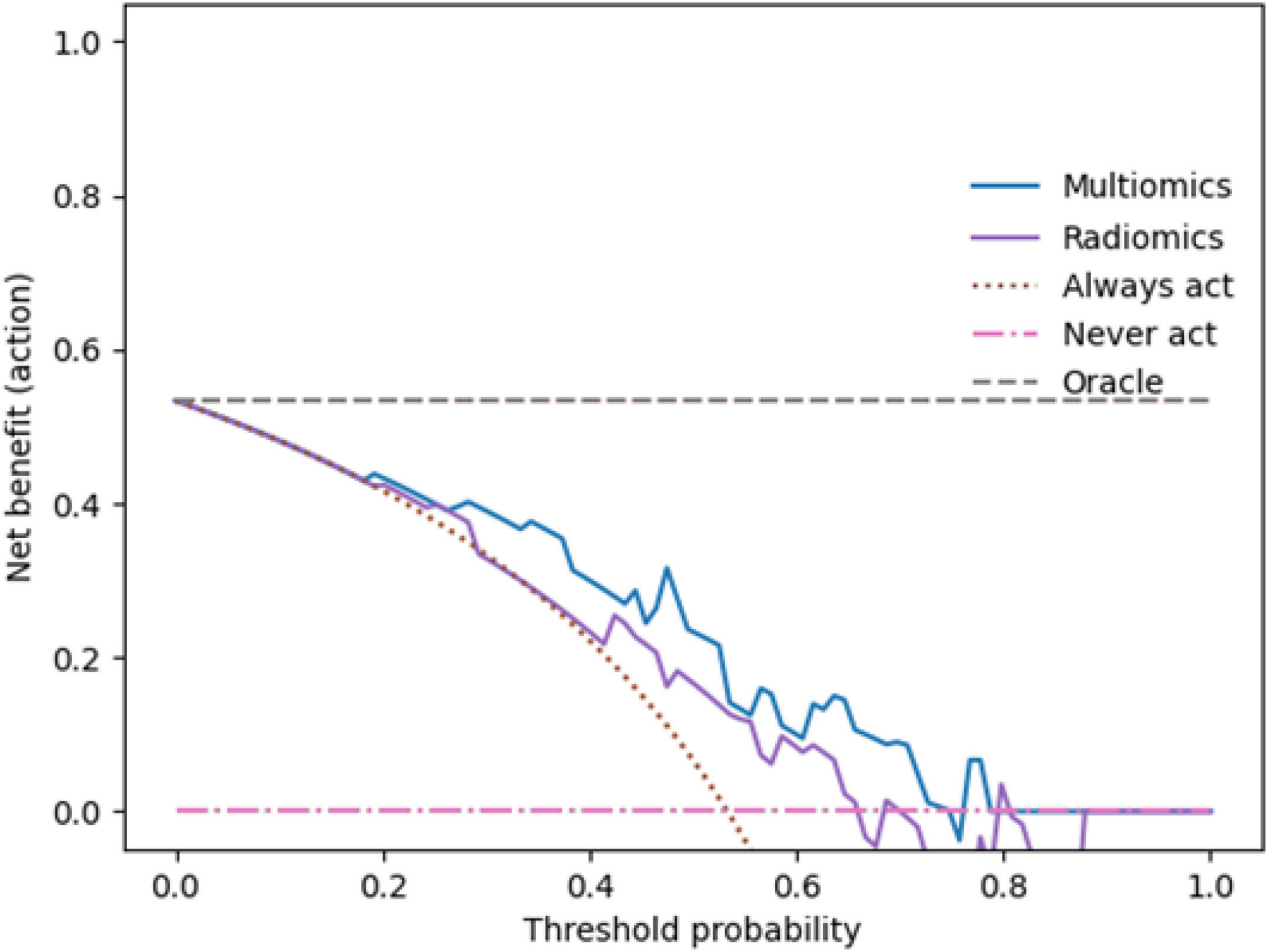
presents the results of the decision curve analysis (DCA) for the combined clinical-radiomics model in Cohort 1. The x-axis indicates the threshold probability, representing the level of risk at which a clinician would initiate treatment, while the y-axis depicts the corresponding net clinical benefit. The blue curve shows the combined model, the purple curve represents the radiomics-only model, the pink curve corresponds to the ‘Treat None’ strategy (assuming no patient has bladder wall invasion), and the grey curve reflects the ‘Treat All’ approach (assuming all patients are affected). The DCA demonstrates that the combined model provides a higher net benefit than the radiomics-only model across a clinically relevant range of threshold probabilities, particularly between 0.39 and 0.65, supporting its potential role in guiding treatment decisions.

**Figure 7:**
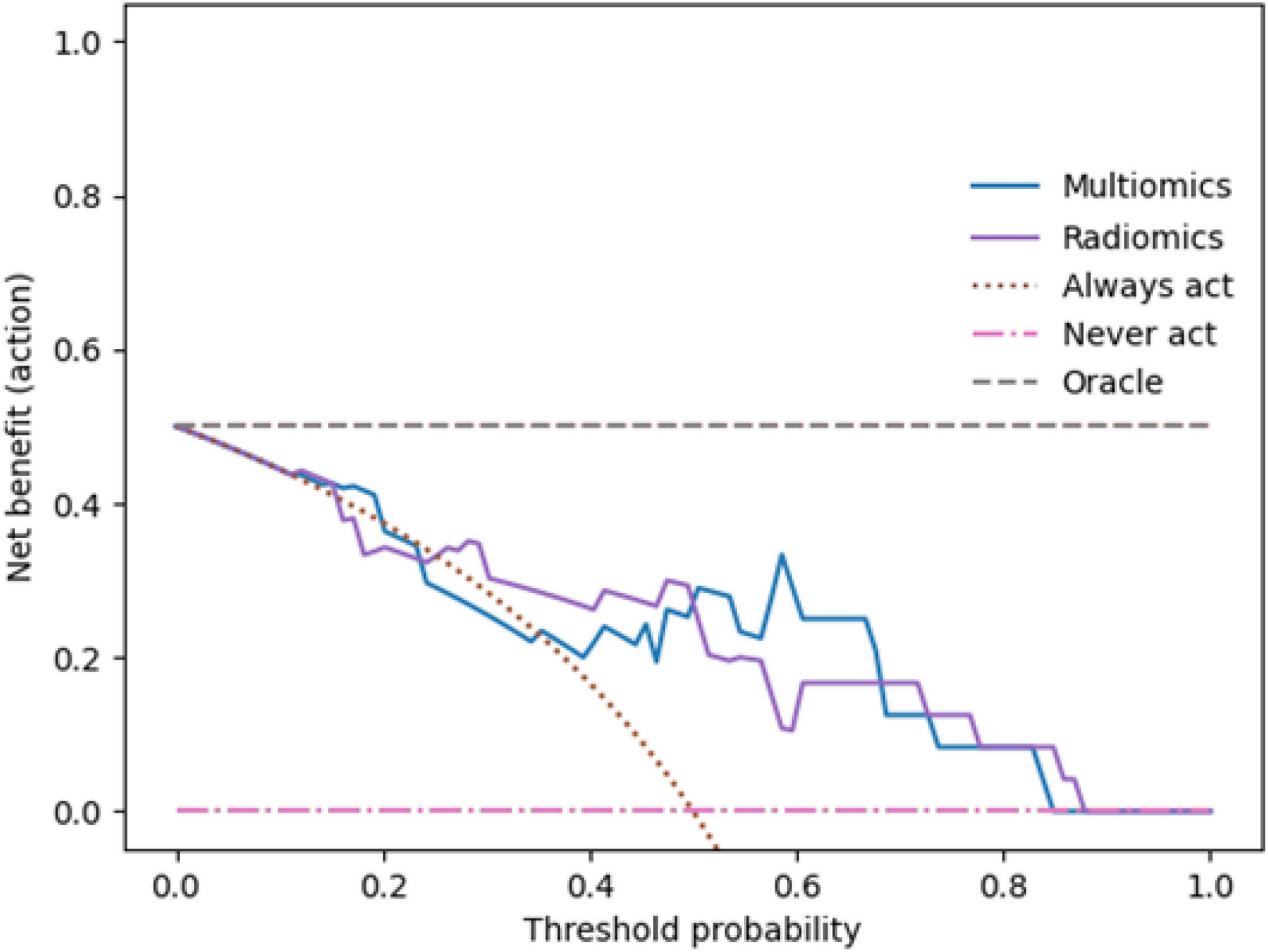
shows the decision curve analysis for the combined and radiomics-only models in Cohort 2. Compared to Cohort 1, the combined model demonstrates an even greater net benefit across a broad threshold range (0.19–0.81), consistent with its superior ROC performance and highlighting its value for individualized treatment decisions.

To evaluate the potential clinical utility of the developed prediction models for assessing bladder wall invasion, we performed decision curve analyses (DCA) for both the radiomics-only and the combined clinical-radiomics models in Cohorts 1 and 2. As shown in **Figures 6** and **7**, the DCA curves indicate that the combined models consistently yield a higher net benefit across a broad range of clinically relevant threshold probabilities, compared to default strategies such as treating all patients (“Always Act”) or none (“Never Act”). This pattern was observed in both validation cohorts, suggesting that the integration of clinical parameters into the radiomics framework enhances the model’s practical applicability. These findings support the potential of the combined model to inform individualized therapeutic decision-making by better aligning diagnostic predictions with clinical risk thresholds.

## Discussion

Accurate preoperative staging of bladder cancer (BCa) is essential for individualized treatment planning and prognostication. According to the European Association of Urology (EAU), tumor stage and grade are key prognostic factors that critically influence therapeutic strategies and the risk of recurrence (51). In particular, differentiating between intravesical (≤T2) and extravesical (≥T3) disease is vital, as extravesical extension is associated with an increased risk of lymphatic spread, distant metastasis, and poor survival. Understaging may lead to undertreatment, while overstaging may expose patients to unnecessary morbidity (51).

Current standard staging relies on transurethral resection of the bladder tumor (TURB), followed by histopathological assessment (52). However, this approach has notable limitations. Tumor heterogeneity and sampling errors can result in underestimation of the true invasion depth. Indeed, up to 50% of patients initially diagnosed with non-muscle-invasive disease (T1) are found to have muscle-invasive cancer (≥T2) at cystectomy (8).

While repeat TURBs may reduce misclassification, they are invasive, associated with increased morbidity, and can delay definitive therapy (53). To address these limitations, clinical guidelines from ESMO and NCCN advocate the use of cross-sectional imaging techniques—primarily CT and MRI—for local staging (4, 6).

These modalities are widely applied to evaluate tumor extent and detect extravesical invasion. However, conventional imaging lacks sufficient accuracy in distinguishing T2 from T3 disease. A meta-analysis reported moderate diagnostic performance, with a pooled sensitivity of 0.71 and specificity of 0.77 for differentiating muscle-invasive from extravesical tumors (54).

Given these challenges, radiomics has emerged as a promising, non-invasive tool to improve staging accuracy. By extracting high-dimensional, quantitative features from standard imaging data, radiomics enables a detailed characterization of tumor morphology, texture, and signal intensity (16–18, 55, 56).

When combined with machine learning, these features can be used to construct predictive models for tumor classification and risk stratification. Previous studies have shown that radiomics-based models can outperform conventional imaging in predicting muscle-invasive disease (57).

However, one clinically relevant factor has remained largely unexplored: the timing of imaging after transurethral resection of the bladder tumor (TURB). Postoperative alterations such as edema, inflammation, or transient bladder wall thickening can affect radiomic feature stability and confound model predictions. To date, no studies have systematically examined how the interval between TURB and imaging influences the performance of CT-based radiomics models for bladder cancer staging.

Our study addresses this gap by evaluating machine learning models based on CT imaging for distinguishing between intravesical (≤T2) and extravesical (≥T3) disease, with particular emphasis on the impact of imaging timing. We developed and validated combined clinical-radiomics models that integrate routinely available laboratory parameters—preoperative creatinine and hemoglobin levels—with radiomic features. These biomarkers have been previously associated with oncologic outcomes in other malignancies (58–60).

To enhance model transparency and mitigate overfitting, we assessed multicollinearity using the Variance Inflation Factor (VIF). Following established guidelines (VIF > 10), we excluded 61 radiomic features, retaining 23 independent variables for model training (32). This filtering strategy ensured a more robust and interpretable feature set.

To further confirm the independence of selected features, we generated heatmaps depicting pairwise Pearson correlations for both radiomics-only and combined models across cohorts. The observed low inter-feature correlations validated the effectiveness of the filtering approach. Together, VIF analysis and correlation heatmaps provided a methodologically sound basis for dimensionality reduction—an essential prerequisite for the clinical translation of radiomics models.

A central focus of our analysis was the comparison between early (<14 days) and delayed (≥14 days) post-TURB imaging cohorts. Our results demonstrate that delayed imaging improves the reproducibility of radiomic features and leads to significantly enhanced staging accuracy. In the delayed cohort, the combined clinical-radiomics model achieved an AUC of 0.82, clearly outperforming radiomics-only models and underscoring the diagnostic value of integrating simple clinical parameters.

These findings suggest that post-surgical changes can adversely affect radiomic data quality, and that imaging timing should be carefully considered in radiomics workflows. In addition to highlighting the benefit of delayed imaging, our study demonstrates that the inclusion of clinical markers substantially improves model performance—an approach that is both cost-effective and readily implementable in clinical practice.

Subgroup analyses by gender and age further elucidated the generalizability of our approach. In male patients, the combined model consistently outperformed the radiomics-only model across both cohorts, with higher sensitivity and specificity. This suggests that routinely available clinical data provide relevant additive prognostic value and that multimodal integration enhances performance in this subgroup.

In contrast, predictive accuracy among female patients was more variable. While the combined model improved results in Cohort 1, it performed less favorably in Cohort 2. This inconsistency may reflect underlying sex-specific differences in tumor biology, inflammatory status, or imaging patterns. Rather than indicating a methodological shortcoming, this variability underscores the potential benefit of sex-specific modeling strategies.

Age-stratified analyses confirmed the added value of clinical integration. The combined model improved performance across both age groups, with notable gains in sensitivity among younger patients (≤70 years) and improved specificity in older individuals (>70 years). These patterns suggest that clinical data provide complementary information across distinct biological and clinical constellations.

Taken together, our results underscore the importance of carefully considering imaging timing, the integration of clinical parameters, and subgroup-specific validation in the design of radiomics-based tools. These strategies may help ensure the development of robust, equitable, and clinically applicable models.

Nonetheless, several limitations must be acknowledged. Our findings are based on a retrospective, single-center cohort, which may limit generalizability. Multicenter prospective validation is required to substantiate these observations. While we focused on CT-based radiomics—given its widespread clinical use—future research should explore MRI-based models and integrate molecular biomarkers to further enhance predictive accuracy. Standardization of imaging protocols and harmonization of radiomic workflows remain crucial for broader clinical adoption.

We also recognize the value of longitudinal analyses, particularly regarding the temporal dynamics of radiomic features in the post-TURB setting. Although our sample size precluded detailed evaluation of individual feature trajectories, future studies should systematically examine such dynamics, potentially using delta-radiomics approaches.

In conclusion, our study demonstrates that a combined clinical-radiomics model—particularly when applied to delayed post-TURB imaging—can significantly enhance the preoperative staging of bladder cancer. The integration of routine laboratory parameters and optimized imaging timing improves model accuracy and supports the development of robust tools for precision diagnostics and individualized treatment planning in uro-oncology. Prospective validation is warranted to confirm these findings and enable clinical translation.

## Data Availability

The data analyzed in this study is subject to the following licenses/restrictions: Ongoing Research. Requests to access these datasets should be directed to catharina.lisson@uni-ulm.de.

## References

[1] Lenis AT Lec PM Chamie K MD MSHS. Bladder cancer: A review. JAMA. (2020) 324:1980–915. doi: 10.1001/jama.2020.17598, PMID: 33201207

[2] Osch FHMv Jochems SHJ van Schooten F-J Bryan RT Zeegers MP. Quantified relations between exposure to tobacco smoking and bladder cancer risk: a meta-analysis of 89 observational studies. Int J Epidemiol. (2016) 45:857–705. doi: 10.1093/ije/dyw044, PMID: 27097748

[3] Burger M Catto JWF Dalbagni G Barton Grossman H Herr H Karakiewicz P. Epidemiology and risk factors of urothelial bladder cancer. Eur Urol. (2013) 63:234–415. doi: 10.1016/j.eururo.2012.07.033, PMID: 22877502

[4] Powles T Bellmunt J Comperat E De Santis M Huddart R Loriot Y. Bladder cancer: ESMO clinical practice guideline for diagnosis, treatment and follow-up☆. Ann Oncol. (2022) 33:244–585. doi: 10.1016/j.annonc.2021.11.012, PMID: 34861372

[5] Flaig TW Spiess PE Agarwal N Bangs R Boorjian SA Buyyounouski MK. Bladder cancer, version 3.2020, NCCN clinical practice guidelines in oncology. J Natl Compr Cancer Netw. (2020) 18:329–545. doi: 10.6004/jnccn.2020.0011, PMID: 32135513

[6] Spiess PE Agarwal N Bangs R Boorjian SA Buyyounouski MK Clark PE. Bladder cancer, version 5.2017, NCCN clinical practice guidelines in oncology. J Natl Compr Cancer Netw. (2017) 15:1240–675. doi: 10.6004/jnccn.2017.0156, PMID: 28982750

[7] Babjuk M Burger M Capoun O Cohen D Compérat EM Dominguez Escrig JL. European Association of Urology guidelines on non–muscle-invasive bladder cancer (Ta, T1, and carcinoma in situ)”. Eur Urol. (2022) 81:75–945. doi: 10.1016/j.eururo.2021.08.010, PMID: 34511303

[8] Ark JT Keegan KA Barocas DA Morgan TM Resnick MJ You C. Incidence and predictors of understaging in patients with clinical T1 urothelial carcinoma undergoing radical cystectomy. BJU Int. (2014) 113:894–995. doi: 10.1111/bju.12245, PMID: 24053444 PMC3874077

[9] Lackner J. Leitlinienreport S3-LL Harnblasenkarzinom Version [3.0] – [Juli] [2024] AWMF-Registernummer: 032/038OL (2024). Available online at: https://www.leitlinienprogramm-onkologie.de/fileadmin/user_upload/Downloads/Leitlinien/Blasenkarzinom/2024-07-31_Leitlinienreport_Harnblasenkarzinom_Konsultationsfassung.pdf (Accessed July 20, 2024).

[10] Lee CH Tan CH Faria SdC Kundra V. Role of imaging in the local staging of urothelial carcinoma of the bladder. Am J Roentgenol. (2017) 208:1193–12055. doi: 10.2214/AJR.16.17114, PMID: 28225635

[11] Obermeyer Z Emanuel EJ. Predicting the future—big data, machine learning, and clinical medicine. New Engl J Med. (2016) 375:12165. doi: 10.1056/NEJMp1606181, PMID: 27682033 PMC5070532

[12] Lambin P Leijenaar RTH Deist TM Peerlings J De Jong EEC Van Timmeren J. Radiomics: the bridge between medical imaging and personalized medicine. Nat Rev Clin Oncol. (2017) 14:749–625. doi: 10.1038/nrclinonc.2017.141, PMID: 28975929

[13] Gillies RJ Kinahan PE Hricak H. Radiomics: images are more than pictures, they are data. Radiology. (2016) 278:5635. doi: 10.1148/radiol.2015151169, PMID: 26579733 PMC4734157

[14] Aerts HJWL Velazquez ER Leijenaar RTH Parmar C Grossmann P Carvalho S. Decoding tumour phenotype by noninvasive imaging using a quantitative radiomics approach. Nat Commun. (2014) 5:1–95. doi: 10.1038/ncomms5006, PMID: 24892406 PMC4059926

[15] Avanzo M Stancanello J El Naqa I. Beyond imaging: The promise of radiomics. Physica Med. (2017) 38:122–39. doi: 10.1016/j.ejmp.2017.05.071, PMID: 28595812

[16] Wu S Zheng J Li Y Yu H Shi S Xie W. A radiomics nomogram for the preoperative prediction of lymph node metastasis in bladder cancerA radiomics nomogram for bladder cancer. Clin Cancer Res. (2017) 23:6904–115. doi: 10.1158/1078-0432.CCR-17-1510, PMID: 28874414

[17] Zhang X Xu X Tian Q Li B Wu Y Yang Z. Radiomics assessment of bladder cancer grade using texture features from diffusion-weighted imaging. J Magnetic Resonance Imaging. (2017) 46:1281–885. doi: 10.1002/jmri.25669, PMID: 28199039 PMC5557707

[18] Choi SJ Park KJ Heo C Park BW Kim M Kim JK. Radiomics-based model for predicting pathological complete response to neoadjuvant chemotherapy in muscle-invasive bladder cancer. Clin Radiol. (2021) 76:6275.e13–627.e21. doi: 10.1016/j.crad.2021.03.001, PMID: 33762138

[19] Zheng Z Xu F Gu Z Yan Y Xu T Liu S. Combining multiparametric MRI radiomics signature with the vesical imaging-reporting and data system (VI-RADS) score to preoperatively differentiate muscle invasion of bladder cancer. Front Oncol. (2021) 11:619893. doi: 10.3389/fonc.2021.619893, PMID: 34055600 PMC8155615

[20] Humphrey PA Moch H Cubilla AL Ulbright TM Reuter VE. The 2016 WHO classification of tumours of the urinary system and male genital organs—Part B: prostate and bladder tumours. Eur Urol. (2016) 70:106–95. doi: 10.1016/j.eururo.2016.02.028, PMID: 26996659

[21] Haralick RM Shanmugam K Dinstein I’H. Textural features for image classification. IEEE Trans Systems Man Cybernet Nr. (1973) 6:610–21. doi: 10.1109/TSMC.1973.4309314

[22] Lambin P Leijenaar RTH Deist TM Peerlings J De Jong EEC Van Timmeren J. Radiomics: the bridge between medical imaging and personalized medicine. Nat Rev Clin Oncol. (2017) 14:749–62. doi: 10.1038/nrclinonc.2017.141, PMID: 28975929

[23] Shen C Liu Z Guan M Song J Lian Y Wang S. 2D and 3D CT radiomics features prognostic performance comparison in non-small cell lung cancer. Trans Oncol. (2017) 10:886–945. doi: 10.1016/j.tranon.2017.08.007, PMID: 28930698 PMC5605492

[24] Zwanenburg A Vallières M Abdalah MA Aerts HJWL Andrearczyk V Apte A. The image biomarker standardization initiative: standardized quantitative radiomics for high-throughput image-based phenotyping. Radiology. (2020) 295:328–85. doi: 10.1148/radiol.2020191145, PMID: 32154773 PMC7193906

[25] Duin RPW Pekalska E. Dissimilarity Representation For Pattern Recognition, The: Foundations And Applications. Singapore: World scientific (2005). Bd. 64.

[26] Sánchez-Maroño N Alonso-Betanzos A Tombilla-Sanromán M. Filter methods for feature selection – A comparative study. In: Yin vH Tino P CorChado E Byrne W Yao X, editors. Intelligent Data Engineering and Automated Learning - IDEAL 2007. Springer, Berlin, Heidelberg (2007). p. 178–87. doi: 10.1007/978-3-540-77226-2_19

[27] Dash M Liu H. Feature selection for classification. Intell Data Anal. (1997) 1:131–565. doi: 10.1016/S1088-467X(97)00008-5

[28] Saeys Y Inza I Larrañaga P. A review of feature selection techniques in bioinformatics. Bioinformatics. (2007) 23:2507–175. doi: 10.1093/bioinformatics/btm344, PMID: 17720704

[29] Breiman L. Random forests. Mach Learn. (2001) 45:5–32. doi: 10.1023/A:1010933404324

[30] Hall M Frank E Holmes G Pfahringer B Reutemann P Witten IH. The WEKA data mining software: an update. ACM SIGKDD Explor Newslett. (2009) 11:10–185. doi: 10.1145/1656274.1656278

[31] Alin A. Multicollinearity. In: Wiley interdisciplinary reviews: computational statistics, vol. 2. (2010). p. 370–74.

[32] Simon L Young D Pardoe I. 10.7—Detecting Multicollinearity Using Variance Inflation Factors. STAT (2018). p. 462.

[33] Daoud JI. Multicollinearity and regression analysis Vol. 949. Bristol, UK: IOP Publishing (2017). p. 012009.

[34] Fitzpatrick BR Mengersen K. A network flow approach to visualising the roles of covariates in random forests. arXiv preprint arXiv:1706.08702. (2017).

[35] Hartmann D Müller D Soto-Rey I Kramer F. Assessing the role of random forests in medical image segmentation. arXiv. (2021). doi: 10.48550/arXiv.2103.16492

[36] Sidey-Gibbons JAM Sidey-Gibbons CJ. Machine learning in medicine: a practical introduction. BMC Med Res Method. (2019) 19:645. doi: 10.1186/s12874-019-0681-4, PMID: 30890124 PMC6425557

[37] Rajkomar A Dean J Kohane I. Machine learning in medicine. New Engl J Med. (2019) 380:1347–585. doi: 10.1056/NEJMra1814259, PMID: 30943338

[38] Parmar C Grossmann P Bussink J Lambin P Aerts HJWL. Machine learning methods for quantitative radiomic biomarkers. Sci Rep. (2015) 5:130875. doi: 10.1038/srep13087, PMID: 26278466 PMC4538374

[39] Laaksonen J Oja E. Classification with learning k-nearest neighbors Vol. 3. Washington, DC. Piscataway, NJ: IEEE (1996) p. 1480–83.

[40] Bansal M Goyal A Choudhary A. A comparative analysis of K-nearest neighbor, genetic, support vector machine, decision tree, and long short term memory algorithms in machine learning. Decision Anal J. (2022) 3:100071. doi: 10.1016/j.dajour.2022.100071

[41] Agrawal T. Hyperparameter optimization using scikit-learn. In: Hyperparameter Optimization in Machine Learning: Make Your Machine Learning and Deep Learning Models More Efficient. Apress, Berkeley, CA (2021). p. 31–51. doi: 10.1007/978-1-4842-6579-6_2

[42] Ahmadinezhad M Arshadi M Hesari E Sharafoddin M Azizi H Khodamoradi F. The relationship between metabolic syndrome and its components with bladder cancer: a systematic review and meta-analysis of cohort studies. Epidemiol Health. (2022) 44. doi: 10.4178/epih.e2022050, PMID: 35638225 PMC9684010

[43] Hoogstraten LMCv Vrieling A van der Heijden AG Kogevinas M Richters A Kiemeney LA. Global trends in the epidemiology of bladder cancer: challenges for public health and clinical practice. Nat Rev Clin Oncol. (2023) 20:287–3045. doi: 10.1038/s41571-023-00744-3, PMID: 36914746

[44] Compérat E Amin MB Cathomas R Choudhury A De Santis M Kamat A. Current best practice for bladder cancer: A narrative review of diagnostics and treatments. Lancet. (2022) 400:1712–215. doi: 10.1016/S0140-6736(22)01188-6, PMID: 36174585

[45] Sun J-W Zhao L-G Yang Y Ma X Wang Y-Y Xiang Y-B. Obesity and risk of bladder cancer: a dose-response meta-analysis of 15 cohort studies. PloS One. (2015) 10:e01193135. doi: 10.1371/journal.pone.0119313, PMID: 25803438 PMC4372289

[46] Lauby-Secretan B Scoccianti C Loomis D Grosse Y Bianchini F Straif K. Body fatness and cancer — Viewpoint of the IARC working group. New Engl J Med. (2016) 375:794–985. doi: 10.1056/NEJMsr1606602, PMID: 27557308 PMC6754861

[47] Connaughton M Dabagh M. Association of hypertension and organ-specific cancer: A meta-analysis. Healthcare. (2022) 10:10745. doi: 10.3390/healthcare10061074, PMID: 35742125 PMC9222904

[48] Gercek O Ulusoy K Yazar VM Topal K. Effects of delayed diagnosis on tumor size, stage and grade in bladder cancer. Int Urol Nephrol. (2024) 56:935–405. doi: 10.1007/s11255-023-03829-1, PMID: 37847325

[49] Pedregosa F Varoquaux G Gramfort A Michel V Thirion B Grisel O. Scikit-learn: machine learning in python. J Mach Learn Res. (2011) 12:2825–30.

[50] van Rossum G Drake FL. Python/C API Manual-Python 2.6. Beaverton, OR. (2009).

[51] Witjes JA Bruins HM Cathomas R Compérat EM Cowan NC Gakis G. European association of urology guidelines on muscle-invasive and metastatic bladder cancer: summary of the 2020 guidelines. Eur Urol. (2021) 79:82–1045. doi: 10.1016/j.eururo.2020.03.055, PMID: 32360052

[52] Ueno Y Takeuchi M Tamada T Sofue K Takahashi S Kamishima Y. Diagnostic accuracy and interobserver agreement for the vesical imaging-reporting and data system for muscle-invasive bladder cancer: a multireader validation study. Eur Urol. (2019) 76:54–565. doi: 10.1016/j.eururo.2019.03.012, PMID: 30922688

[53] Panebianco V Narumi Y Barchetti G Montironi R Catto JWF. Should we perform multiparametric magnetic resonance imaging of the bladder before transurethral resection of bladder? Time to reconsider the rules. Eur Urol. (2019) 76:57–585. doi: 10.1016/j.eururo.2019.03.046, PMID: 31000355

[54] Gandhi N Krishna S Booth CM Breau RH Flood TA Morgan SC. Diagnostic accuracy of magnetic resonance imaging for tumour staging of bladder cancer: systematic review and meta-analysis. BJU Int. (2018) 122:744–535. doi: 10.1111/bju.14366, PMID: 29727910

[55] Cha KH Hadjiiski L Chan H-P Weizer AZ Alva A Cohan RH. Bladder cancer treatment response assessment in CT using radiomics with deep-learning. Sci Rep. (2017) 7:87385. doi: 10.1038/s41598-017-09315-w, PMID: 28821822 PMC5562694

[56] Cacciamani GE Nassiri N Varghese B Maas M King KG Hwang D. Radiomics and bladder cancer: current status. Bladder Cancer. (2020) 6:343–625. doi: 10.3233/BLC-200293

[57] Kozikowski M Suarez-Ibarrola R Osiecki R Bilski K Gratzke C Shariat SF. Role of radiomics in the prediction of muscle-invasive bladder cancer: A systematic review and meta-analysis. Eur Urol Focus. (2022) 8:728–385. doi: 10.1016/j.euf.2021.05.005, PMID: 34099417

[58] Obermair A Handisurya A Kaider A Sevelda P Kölbl H Gitsch G. The relationship of pretreatment serum hemoglobin level to the survival of epithelial ovarian carcinoma patients: A prospective review. Cancer. (1998) 83:726–315. doi: 10.1002/(SICI)1097-0142(19980815)83:4<726::AID-CNCR14>3.0.CO;2-U, PMID: 9708937

[59] Hamai Y Hihara J Taomoto J Yamakita I Ibuki Y Okada M. Hemoglobin level influences tumor response and survival after neoadjuvant chemoradiotherapy for esophageal squamous cell carcinoma. World J Surg. (2014) 38:15. doi: 10.1007/s00268-014-2486-2, PMID: 24615604

[60] Lafleur J Hefler-Frischmuth K Grimm C Schwameis R Gensthaler L Reiser E. Prognostic value of serum creatinine levels in patients with epithelial ovarian cancer. Anticancer Res. (2018) 38:5127–305. doi: 10.21873/anticanres.12834, PMID: 30194159

