# Supplementary material for: Machine learning-based radiomics for bladder cancer staging: evaluating the role of imaging timing in differentiating T2 from T3 disease
Source: Front Oncol. 2025 Sep 26;15:1591742. doi: 10.3389/fonc.2025.1591742 (PMC12512170; doi:10.3389/fonc.2025.1591742)
Supplement: Supplementary file 1 [file DataSheet1.docx]

# SUPPLEMENTAL MATERIALS

## Supplemental Table S1: Settings of the Radiomics Feature Extraction

**Settings of the Radiomics Feature Extraction**

ResampleFilter 1

ResampleSpacingX 1

ResampleSpacingY 1

ResampleSpacingZ 1

BinMethod FBS

BinAmount 20

LoGFilter 0

LoGSigma 1

SecondOrderDistance 1

MatrixAggregationMethod 3D average directions (3D:avg)

## Supplemental Table S2: Image Biomarker Parameters

**Image Biomarker Computation / Parameters**

**Basic Features**

Short axis

Long axis

Volume

Density median

Density

**Radiomics Features of First Order:**

**Intensity-Based Features**

Intensity Variation

Intensity Energy

Intensity Interquartile Range

Intensity Kurtosis

Intensity Max

Intensity Mean

Intensity Mean absolute deviation

Intensity Median absolute deviation

Intensity Min

Intensity 10th percentile

Intensity 25th percentile

Intensity 50th percentile

Intensity 75th percentile

Intensity 90th percentile

Intensity Quartile coefficient dispersion

Intensity Range

Intensity Robust mean absolute deviation

Intensity Root mean square

Intensity Skewness

Intensity SD

Intensity Variance

**Intensity Histogram Features**

Histogram Coefficient variation

Histogram Entropy

Histogram Interquartile range

Histogram Kurtosis

Histogram Max

Histogram Maximum histogram gradient

Histogram Maximum histogram gradient intensity

Histogram Mean

Histogram Mean abs deviation

Histogram Median abs deviation

Histogram Min

Histogram Minimum histogram gradient

Histogram Minimum histogram gradient intensity

Histogram 10th percentile

Histogram 25th percentile

Histogram 50th percentile

Histogram 75th percentile

Histogram 90th percentile

Histogram Quartile coefficient dispersion

Histogram Range

Histogram Robust mean abs deviation

Histogram Skewness

Histogram SD

Histogram Uniformity

Histogram Variance

**Radiomics Features of Second Order:**

**Gray Level Co-Occurrence Matrix**

**(GLCM) Features**

GLCM Angular second moment

GLCM Auto correlation

GLCM Cluster prominence

GLCM Cluster shade

GLCM Cluster tendency

GLCM Contrast

GLCM Correlation

GLCM Difference average

GLCM Difference entropy

GLCM Difference variance

GLCM Dissimilarity

GLCM Information correlation 1

GLCM Information correlation 2

GLCM Inverse difference

GLCM Inverse difference moment

GLCM Inverse difference moment normalized

GLCM Inverse difference normalized

GLCM Inverse variance

GLCM Joint average

GLCM Joint entropy

GLCM Joint maximum

GLCM Joint variance

GLCM Standard deviation

GLCM Sum of averages

GLCM Sum of entropy

GLCM Sum of variance

Radiomic feature extraction was performed by systematically analyzing grey-level intensity patterns within the defined volumes of interest (VOIs). To ensure methodological consistency and reproducibility, all texture features were computed in accordance with the standardized definitions and computational frameworks outlined by the Image Biomarker Standardisation Initiative (IBSI) (1).

## Supplemental Table S3: ViF Analysis Radiomics

**Features with high ViF (Variance inflation factor) – Excluded**

**Exclude**

## Dropping 'Histogram Min - Value' with VIF = inf

## Dropping 'Histogram Max - Value' with VIF = inf

## Dropping 'Histogram 25th percentile - Value' with VIF = inf

## Dropping 'GLCM Cluster tendency - Value' with VIF = inf

## Dropping 'GLCM Difference average - Value' with VIF = inf

## Dropping 'Intensity Range - Value' with VIF = 75059993789508.27

## Dropping 'GLCM Sum of averages - Value' with VIF = 127736325477.79

## Dropping 'GLCM Joint variance - Value' with VIF = 5571772488.87

## Dropping 'Intensity 75th percentile - Value' with VIF = 44528620.03

## Dropping 'Intensity Min - Value' with VIF = 12256192.25

## Dropping 'GLCM Inverse difference - Value' with VIF = 10602052.29

## Dropping 'Histogram Variance - Value' with VIF = 1329432.23

## Dropping 'Histogram Mean - Value' with VIF = 213997.96

## Dropping 'GLCM Dissimilarity - Value' with VIF = 188549.99

## Dropping 'GLCM Standard deviation - Value' with VIF = 122525.55

## Dropping 'Intensity Mean absolute deviation - Value' with VIF = 80953.55

## Dropping 'Histogram SD - Value' with VIF = 46133.21

## Dropping 'Intensity Mean - Value' with VIF = 38971.96

## Dropping 'Intensity SD - Value' with VIF = 24976.45

## Dropping 'GLCM Joint entropy - Value' with VIF = 22466.43

## Dropping 'Histogram 10th percentile - Value' with VIF = 21485.74

## Dropping 'GLCM Joint average - Value' with VIF = 14018.64

## Dropping 'Histogram Entropy - Value' with VIF = 10804.62

## Dropping 'Histogram Kurtosis - Value' with VIF = 8881.14

## Dropping 'Intensity Median absolute deviation - Value' with VIF = 7783.74

## Dropping 'Intensity Robust mean absolute deviation - Value' with VIF = 7291.42

## Dropping 'GLCM Contrast - Value' with VIF = 5968.28

## Dropping 'Intensity 25th percentile - Value' with VIF = 5560.72

## Dropping 'GLCM Cluster prominence - Value' with VIF = 4215.78

## Dropping 'Intensity Root mean square - Value' with VIF = 2844.42

## Dropping 'Histogram Range - Value' with VIF = 2499.70

## Dropping 'Histogram Skewness - Value' with VIF = 2401.31

## Dropping 'Intensity Variation - Value' with VIF = 2037.90

## Dropping 'GLCM Difference entropy - Value' with VIF = 1943.44

## Dropping 'Histogram 75th percentile - Value' with VIF = 1770.03

## Dropping 'Intensity Variance - Value' with VIF = 1557.55

## Dropping 'Histogram 90th percentile - Value' with VIF = 1333.01

## Dropping 'Histogram Uniformity - Value' with VIF = 1170.49

## Dropping 'Histogram Maximum histogram gradient - Value' with VIF = 976.84

## Dropping 'Histogram Minimum histogram gradient intensity - Value' with VIF = 893.16

## Dropping 'Intensity 90th percentile - Value' with VIF = 851.32

## Dropping 'Histogram 50th percentile - Value' with VIF = 696.41

## Dropping 'GLCM Sum of entropy - Value' with VIF = 638.54

## Dropping 'Histogram Median abs deviation - Value' with VIF = 573.83

## Dropping 'Histogram Maximum histogram gradient intensity - Value' with VIF = 286.21

## Dropping 'Density median - Value' with VIF = 247.38

## Dropping 'GLCM Inverse difference normalized - Value' with VIF = 204.41

## Dropping 'GLCM Inverse difference moment - Value' with VIF = 174.02

## Dropping 'Histogram Mean abs deviation - Value' with VIF = 139.72

## Dropping 'Volume - Value' with VIF = 127.78

## Dropping 'Intensity 10th percentile - Value' with VIF = 104.29

## Dropping 'GLCM Difference variance - Value' with VIF = 70.90

## Dropping 'GLCM Joint maximum - Value' with VIF = 52.25

## Dropping 'Histogram Coefficient variation - Value' with VIF = 37.08

## Dropping 'GLCM Information correlation 2 - Value' with VIF = 30.10

## Dropping 'Intensity Energy - Value' with VIF = 21.11

## Dropping 'Intensity Interquartile Range - Value' with VIF = 19.84

## Dropping 'Intensity Kurtosis - Value' with VIF = 18.21

## Dropping 'Intensity Skewness - Value' with VIF = 15.38

## Dropping 'GLCM Correlation - Value' with VIF = 14.29

**Features with low ViF (Variance inflation factor) – Retained**

**Re**

## Histogram Interquartile range – Value = 7.544764

## Histogram Minimum histogram gradient – Value = 8.251255

## Histogram Quartile coefficient dispersion – Value= 8.016959

## Histogram Robust mean abs deviation – Value = 5.128227

## Intensity Max – Value = 2.846662

## Intensity 50th percentile - Value = 2.941639

## Intensity Quartile coefficient dispersion – Value = 3.943631

## GLCM Angular second moment – Value = 6.908069

## GLCM Auto correlation - Value = 4.948483

## GLCM Cluster shade – Value = 6.161783

## GLCM Information correlation 1 - Value = 2.048208

## GLCM Inverse difference moment normalized – Value = 5.005999

## GLCM Inverse variance - Value = 2.715986

## GLCM Sum of variance - Value = 5.309781

## Long axis - Value = 5.482701

## Short axis - Value = 4.495761

## Supplemental Table S4: ViF Analysis Multiomics

**Features with high ViF (Variance inflation factor) – Excluded**

**Exclude**

Dropping 'Histogram Min - Value' with VIF = inf

Dropping 'Histogram Max - Value' with VIF = inf

Dropping 'GLCM Cluster tendency - Value' with VIF = inf

Dropping 'Histogram 25th percentile - Value' with VIF = inf

Dropping 'GLCM Difference average - Value' with VIF = 4503599627370496.00

Dropping 'Intensity Range - Value' with VIF = 104734875055127.81

Dropping 'GLCM Joint average - Value' with VIF = 149417724274.92

Dropping 'GLCM Joint variance - Value' with VIF = 8075113167.19

Dropping 'Intensity 75th percentile - Value' with VIF = 63285217.87

Dropping 'GLCM Inverse difference - Value' with VIF = 15576357.96

Dropping 'Intensity Min - Value' with VIF = 14504131.61

Dropping 'Histogram Variance - Value' with VIF = 1667469.72

Dropping 'Histogram Mean - Value' with VIF = 292378.45

Dropping 'GLCM Dissimilarity - Value' with VIF = 230450.51

Dropping 'GLCM Standard deviation - Value' with VIF = 132662.10

Dropping 'Intensity Mean absolute deviation - Value' with VIF = 89221.74

Dropping 'Histogram SD - Value' with VIF = 54765.57

Dropping 'Intensity Mean - Value' with VIF = 46112.02

Dropping 'Histogram 10th percentile - Value' with VIF = 37436.53

Dropping 'GLCM Joint entropy - Value' with VIF = 26694.95

Dropping 'Intensity SD - Value' with VIF = 24904.06

Dropping 'GLCM Sum of averages - Value' with VIF = 15692.64

Dropping 'Histogram Entropy - Value' with VIF = 12303.40

Dropping 'Intensity Median absolute deviation - Value' with VIF = 10210.31

Dropping 'Histogram Kurtosis - Value' with VIF = 9867.34

Dropping 'Intensity Robust mean absolute deviation - Value' with VIF = 8586.34

Dropping 'GLCM Contrast - Value' with VIF = 6952.18

Dropping 'Intensity 25th percentile - Value' with VIF = 6302.83

Dropping 'GLCM Cluster prominence - Value' with VIF = 4809.39

Dropping 'GLCM Difference entropy - Value' with VIF = 3205.79

Dropping 'Histogram Range - Value' with VIF = 2987.66

Dropping 'Intensity Variance - Value' with VIF = 2689.30

Dropping 'Histogram Skewness - Value' with VIF = 2323.80

Dropping 'Intensity Variation - Value' with VIF = 2251.96

Dropping 'Histogram 75th percentile - Value' with VIF = 2243.66

Dropping 'Intensity Root mean square - Value' with VIF = 2014.73

Dropping 'Histogram 90th percentile - Value' with VIF = 1480.94

Dropping 'Histogram Uniformity - Value' with VIF = 1293.03

Dropping 'Histogram Maximum histogram gradient - Value' with VIF = 1192.37

Dropping 'Histogram Minimum histogram gradient intensity - Value' with VIF = 1018.44

Dropping 'Intensity 90th percentile - Value' with VIF = 1006.45

Dropping 'GLCM Sum of entropy - Value' with VIF = 758.07

Dropping 'Histogram 50th percentile - Value' with VIF = 715.93

Dropping 'Histogram Mean abs deviation - Value' with VIF = 606.67

Dropping 'Histogram Maximum histogram gradient intensity - Value' with VIF = 305.70

Dropping 'Density median - Value' with VIF = 263.63

Dropping 'GLCM Inverse difference normalized - Value' with VIF = 214.47

Dropping 'GLCM Inverse difference moment - Value' with VIF = 179.84

Dropping 'Volumen - Value' with VIF = 169.17

Dropping 'Histogram Median abs deviation - Value' with VIF = 153.10

Dropping 'Intensity 10th percentile - Value' with VIF = 127.92

Dropping 'GLCM Difference variance - Value' with VIF = 76.67

Dropping 'GLCM Joint maximum - Value' with VIF = 64.47

Dropping 'Histogram Coefficient variation - Value' with VIF = 38.19

Dropping 'GLCM Information correlation 2 - Value' with VIF = 33.36

Dropping 'Intensity Energy - Value' with VIF = 23.92

Dropping 'Intensity Interquartile Range - Value' with VIF = 22.58

Dropping 'Intensity Kurtosis - Value' with VIF = 20.48

Dropping 'Intensity Skewness - Value' with VIF = 17.60

Dropping 'GLCM Correlation - Value' with VIF = 15.32

**Features with low ViF (Variance inflation factor) – Retained**

**Re**

BMI in kg/m² = 1.558104

Smoker? 0=never 1=former 2=current = 1.293051

Arterial Hypertension 1= yes 0=no = 1.566585

Renal insufficiency 1=yes 0=no = 1.613859

Diabetes mellitus 1= yes 0=no = 1.305722

pre-op Krea µmol/l = 1.742977

pre-op Hb in g/dl = 1.602616

Histogram Interquartile range – Value = 8.474993

Histogram Minimum histogram gradient - Value = 8.581509

Histogram Quartile coefficient dispersion – Value = 9.128829

Histogram Robust mean abs deviation - Value = 6.196623

Intensity Max - Value = 3.025878

Intensity 50th percentile - Value = 3.012444

Intensity Quartile coefficient dispersion – Value = 4.193156

GLCM Angular second moment - Value = 8.587290

GLCM Auto correlation - Value = 5.585194

GLCM Cluster shade - Value = 6.518089

GLCM Information correlation 1 - Value = 2.490897

GLCM Inverse difference moment normalized – Value = 5.270654

GLCM Inverse variance - Value = 3.050269

GLCM Sum of variance - Value = 5.929919

Long axis - Value = 5.881157

Short axis - Value = 5.317313

## Supplemental Table S5: Model Development Details

**Model Development and Computational Setup**

| Aspect | Details |
| --- | --- |
| Model Optimization | Grid search procedure used to fine-tune hyperparameters (2). |
| Optimal Parameters | `max_depth = 8`, `criterion = 'gini'`. |
| Validation Method | Fivefold cross-validation to ensure robustness. |
| Clinical Parameters Included | Smoking status, arterial hypertension, diabetes mellitus, renal insufficiency, preoperative creatinine, preoperative hemoglobin and tumor size. |
| Software & Implementation | Feature selection and model construction were implemented using the open-source Python machine learning library Scikit-learn (Python version 3.10, Scikit-learn version 0.23.3, http://scikit-learn.org/) |

# References:

1. Zwanenburg A, Vallières M, Abdalah MA, Aerts HJ, Andrearczyk V, Apte A, et al. The image biomarker standardization initiative: standardized quantitative radiomics for high-throughput image-based phenotyping. Radiology. 2020;295(2):328–38.

2. Agrawal T. Hyperparameter Optimization Using Scikit-Learn. In: Hyperparameter Optimization in Machine Learning: Make Your Machine Learning and Deep Learning Models More Efficient [Internet]. Berkeley, CA: Apress; 2021. p. 31–51. Available from: https://doi.org/10.1007/978-1-4842-6579-6_2
